# Host-Feeding Preference of the Mosquito, *Culex quinquefasciatus*, in Yucatan State, Mexico

**DOI:** 10.1673/031.010.3201

**Published:** 2010-04-06

**Authors:** Julian E. Garcia-Rejon, Bradley J. Blitvich, Jose A. Farfan-Ale, Maria A. Loroño-Pino, Wilberth A. Chi Chim, Luis F. Flores-Flores, Elsy Rosado-Paredes, Carlos Baak-Baak, Jose Perez-Mutul, Victor Suarez-Solis, Ildefonso Fernandez-Salas, Barry J. Beaty

**Affiliations:** ^1^ Laboratorio De Arbovirología, Centro de Investigaciones Regionales Dr. Hideyo Noguchi, Universidad Autónoma de Yucatan, Ave. Itzáes No. 490 × 59, Centro Mérida, Yucatán, México. 97000.; ^2^ Department of Veterinary Microbiology and Preventive Medicine, Iowa State University. 2116 Veterinary Medicine Building. Ames, Iowa, USA 50011-1250; ^3^ Departamento de Neurociencias, Centro de Investigaciones Regionales Dr. Hideyo Noguchi, Universidad Autónoma de Yucatán, Ave. Itzáes No. 490 × 59, Centro Mérida, Yucatán, México. 97000.; ^4^ Unidad Interinstitucional de Investigación Clínica y Epidemiológica. Facultad de Medicina, Universidad Autónoma de Yucatán Av. Itzáes No. 498 × 59-A Centro Mérida, Yucatán, México. 97000.; ^5^ Laboratorio de Entomología Médica, Facultad de Ciencias Biológicas, Universidad Autónoma de Nuevo León, Ap. Postal 109-F, San Nicolás de los Garza, Nuevo León, México.; ^6^ Arthropod-borne and Infectious Disease Laboratory, Department of Microbiology, Immunology and Pathology, College of Veterinary Medicine and Biomedical Science, Colorado State University, 3185 Rampart Road, Fort Collins, Colorado, USA. 80523-1692.

**Keywords:** Blood meal identification, flavivirus, human blood index, West Nile virus

## Abstract

Studies were conducted to determine the host-feeding preference of *Culex quinquefasciatus* Say (Diptera: Culicidae) in relation to the availability of human and domestic animals in the city of Merida, Yucatan State, Mexico. Mosquitoes were collected in the backyards of houses using resting wooden boxes. Collections were made five times per week from January to December 2005. DNA was extracted from engorged females and tested by PCR using universal avian- and mammalian-specific primers. DNA extracted from avian-derived blood was further analyzed by PCR using primers that differentiate among the birds of three avian orders: Passeriformes, Columbiformes and Galliformes. PCR products obtained from mammalian-derived blood were subjected to restriction enzyme digestion to differentiate between human-, dog-, cat-, pig-, and horse-derived blood meals. Overall, 82% of engorged mosquitoes had fed on birds, and 18% had fed on mammals. The most frequent vertebrate hosts were Galliformes (47.1%), Passeriformes (23.8%), Columbiformes (11.2%) birds, and dogs (8.8%). The overall human blood index was 6.7%. The overall forage ratio for humans was 0.1, indicating that humans were not a preferred host for *Cx. quinquefasciatus* in Merida.

## Introduction

Knowledge of the blood-feeding preferences of a mosquito species provides important insight into the dynamics of virus transmission and allows vector control authorities to design and implement efficient strategies for vector control (Tempelis 1975; Vinogradova 2000). The significance of *Culex quinquefasciatus* Say (Diptera: Culicidae) in the transmission of West Nile virus (*Flaviviridae*: *Flavivirus*) (WNV) in Mexico is poorly understood. However, it is well documented that this species is a principal vector of WNV in the United States (Turell et al. 2005). *Cx. quinquefasciatus* has accounted for more than half of the WNV-infected mosquito pools reported during some transmission seasons in the United States (Hayes et al. 2005).

*Cx. quinquefasciatus* populations from the United States feed readily on both birds and mammals (Reisen and Reeves 1990; Reisen et al 1990; Zinser et al. 2004). For instance, in Tucson, Arizona, 50% of engorged *Cx. quinquefasciatus* had fed on humans, 32% had fed on birds, and 12% had fed on dogs (Zinser et al. 2004). *Cx. quinquefasciatus* populations in East Baton Rouge Parish, Louisiana fed most frequently on dogs (69%), followed by birds (16%) and humans (11%) (Niebylski and Meek 1992). In Harris County, Texas, 52%) of engorged *Cx. quinquefasciatus* had fed exclusively on mammals, and 39% had fed exclusively on birds; the remainder contained mixed avian and mammalian blood meals (Molaei et al. 2007). Dogs were the most common (41%) vertebrate host followed by mourning doves (18%) and cats (9%). *Cx. quinquefasciatus* populations from the United States are relatively efficient laboratory vectors of WNV under laboratory conditions (Goddard et al. 2002; Turell et al. 2005). Taken together, these findings suggest that *Cx. quinquefasciatus* serves both as an important amplification and as a bridging vector of WNV in the United States.

Serologic evidence has demonstrated widespread WNV activity in Mexico (Blitvich et al. 2003; Estrada-Franco et al. 2003; Farfan-Ale et al. 2004, 2006; Loroño et al 2003). The seroprevalence for WNV in horses sampled in Yucatan State in 2002 was 1.2% (Loroño et al. 2003). In a longitudinal study of WNV infection in birds conducted in Yucatan State from 2000 to 2003, the seroprevalence for WNV was 0.06% (Farfan-Ale et al. 2004). In a more recent study, 52% of horses sampled in the neighboring state of Quintana Roo in 2003 were seropositive for WNV (Farfan-Ale et al. 2006). Several WNV isolates have also been collected in Mexico. One isolate was from a pool of *Cx. quinquefasciatus* collected in northern Mexico in 2003 (Elizondo-Quiroga et al. 2005); all other WNV isolates were from sick or dead vertebrates (Beasley et al 2004; Blitvich et al. 2004; Deardorff et al 2006; Elizondo-Quiroga et al. 2005; Estrada-Franco et al. 2003). This study was conducted to determine the host-feeding preferences of *Cx. quinquefasciatus* in an urban area of Yucatan State, Mexico because WNV has been isolated from *Cx. quinquefasciatus* in Mexico and serves as an important ampiflying host and a bridging vector of WNV in the United States.

## Materials and Methods

### Description of study sites

Mosquito collections were made in the city of Merida (20° 58′ 12″ N, 89° 37′ 12″ W) in Yucatan State, Mexico. Merida has a warm and humid climate throughout the year with an average annual temperature of 26°C (79°F) and distinct seasons, rainy from May to October and dry from November to April. The average annual precipitation is 929 mm (766 mm in the rainy season, 163 mm in the dry season). The average elevation in Merida is 10 meters. The population is approximately 750,000.

### Mosquito collections

Diurnal outdoor resting mosquitoes were collected in the backyards of 40 houses. The houses were located in a 36 square-mile area of Merida. The houses were spaced evenly apart; each was approximately 0.5 to 1 mile from the closest study site. Mosquitoes were collected from each backyard five times per week from January 8, 2005 to December 22, 2005. Collections were made using resting wooden boxes (0.5 m^3^) painted red on both the inside and outside (Reisen and Pfunter 1987). One resting box was placed in each backyard. Resting boxes were placed on the ground in a sheltered position facing west to prevent morning sunlight from entering. Mosquitoes were removed from resting boxes between 0600 and 0900 hours using handheld, battery-operated aspirators. Mosquitoes were transported alive to the laboratory, euthanized in a -70°C freezer, and then identified on chill tables according to species, sex, and blood feeding status using morphological characteristics (Carpenter and LaCasse 1955; Darsie and Ward 1981).

### Census of vertebrate hosts

A census was taken of humans and domestic animals (birds and mammals) at each site. This was done by interviewing the occupants of each residence. If the number of vertebrate animals at a site changed (i.e. due to death), the site was no longer used for this study. Sites were only used if the number and species composition of vertebrates remained constant during the study period. Due to practical constraints, the numbers of freeranging birds temporally and spatially associated with each study site were not estimated. The numbers of humans and domestic vertebrates in neighboring houses were not counted.

### Blood meal identification

Abdomens were removed from engorged females and individually placed into 1.5 ml eppendorf tubes. Abdomens were manually homogenized in 600 ul of phosphate-buffered saline (pH 7.4) using a sterile micro-pestle. Homogenates were applied to QIAshredder spin columns (Qiagen, www.qiagen.com) and centrifuged (14,000 g for 3 minutes at 4° C). DNA was extracted from supernatants using the QIAamp DNA extraction kit following the manufacturer's instructions. Extracted DNA was analyzed by PCR using universal avian-and mammalian-specific primers that amplify 508 and 772-nt fragments, respectively, of the mitochondrial cytochrome b gene (Ngo and Kramer 2003; Cicero and Johnson 2001). DNA from each avian-derived blood meal was further examined in a single PCR reaction using 3 pairs of order-specific primers that differentiate among Passeriformes, Galliformes and Columbiformes; these primers amplify 165, 210 and 333-nt fragments, respectively, of the mitochondrial cytochrome b gene. PCR products derived from mammalian blood meals were subjected to restriction enzyme analysis using Alu I to differentiate between human-, dog-, cat-, pig-and horse-derived blood meals (Zehner et al 1998). The performance of the restriction enzyme assay was validated using DNA extracted from the blood of known vertebrate species. Species used for the validation experiments were: chicken (Galliformes), rock pigeon (Columbiformes), Yucatan jay (Passeriformes), human, dog, cat, horse and pig. Prior to the analysis of field-caught mosquitoes, the specificity of each primer pair was validated by PCR using DNA from the above mentioned vertebrate species. Each primer pair produced a PCR product of the expected size when tested with DNA from its intended target(s). Nucleotide sequencing confirmed that the amplified products were indeed mitochondrial cytochrome b DNA and that the expected Alu I restriction enzyme sites were present (Genbank Accession numbers FJ160756, FJ160757, FJ160758, FJ160759, FJ160760, FJ160761, FJ160762 and FJ160763) None of the primer pairs generated a detectable PCR product when tested with DNA from non-target species (data not shown).

### Data analysis

The human blood index (HBI), which is defined as the proportion of freshly engorged mosquitoes containing human blood, was calculated as described by Garrett-Jones (1964). The forage ratio (FR), which quantifies vector selection of a particular vertebrate host rather than other available hosts, was also measured (Boreham and Garrett-Jones 1973). FRs were calculated by determining the percent of *Cx. quinquefasciatus* females containing blood of a particular host, divided by the percent of the total available host population represented by that particular host (Hess et al. 1968). An FR of 1.0 indicates neither a selective bias nor avoidance of a particular host animal; FRs significantly > 1.0 indicate a selective bias, and values < 1.0 indicate avoidance of a host in favor of other available hosts.

The host feeding index (HFI), which is defined as the observed proportion of feeds on one host with respect to another divided by the expected comparative proportion of feeds on these two hosts, was calculated using the formula described by Richards et al. (2006). The formula is as follows:

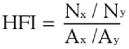

where N_x_ and N_y_ are the mean numbers of blood meals taken from hosts x and y per study site, respectively, and A_x_ and A_y_ are the mean numbers of hosts x and y per study site, respectively. An index of 1.0 indicates equal feeding on the two hosts. Results < 1.0 and > 1.0 indicate a decrease or increase, respectively, in feeding on the first host relative to the second. HFIs were calculated for each pair of hosts. One advantage of the HFI versus the FR is that it does not require a full animal census (Kay et al. 1979)

## Results and Discussion

### Mosquito collections

A total of 4,644 *Cx. quinquefasciatus* were captured in this study (Table 1). Of these, 1600 were female, and 3,044 were male. A total of 658 females were classified as engorged, 233 as gravid, and 709 did not contain blood. *Cx. quinquefasciatus* were present year-round, but they were most abundant in August (17.4% of the total collection was made at this time) and November (15.7%).

### Hood feeding preferences

**Figure 1.**
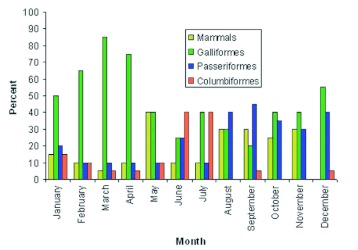
The source of blood meals for engorged *Culex quinquefasciatus* captured outdoors, in the backyards of houses, each month in Merida, Yucatan State. DNA was extracted from engorged mosquitoes and analyzed by PCR using universal avian-and mammalian-specific primers. DNA extracted from avian-derived blood meals was further examined by PCR using primers that differentiate between Passeriformes, Columbiformes and Galliformes. Data are presented as the percentage of mosquitoes containing blood derived from mammals, avians, Passeriformes, Columbiformes and Galliformes each month. High quality figures are available online.

For the blood meal identification experiments, 240 engorged females were used (20 per month). Overall, 197 (82.1%) mosquitoes contained avian-derived blood meals and 43 (17.9%)) contained mammalian-derived blood meals (Figure 1). The proportion of mosquitoes containing avian-derived blood each month ranged from 60% (May) to 100% (December). No mosquitoes contained mixed blood meals. Forty percent of the DNA samples failed to yield a detectable PCR product. By comparison, 27% of field-collected engorged *Culiseta* and *Anopheles* spp. mosquitoes from New York did not yield a detectable PCR product (Ngo and Kramer 2003). Additional mosquitoes were analyzed when PCR negative samples were encountered, until the monthly total of PCR positive samples reached 20.

The most frequent vertebrate hosts for *Cx. quinquefasciatus* were Galliformes (47.1%), Passeriformes (23.8%) and Columbiformes (11.2%) (Table 2). These findings suggest that *Cx. quinquefasciatus* populations in Merida are strongly ornithophilic. The proportion of mosquitoes that had acquired blood meals from galliformes each month ranged from 20% (September) to 85% (March) (Figure 1). The proportion of mosquitoes that had acquired blood meals from passeriformes also exhibited considerable seasonal variation, as this avian order was fed upon more frequently in the latter half of the year. From January to July, the proportion of mosquitoes that had acquired blood meals from passeriformes each month ranged from 10% to 25% (mean: 13.6%). From August to December, the proportion of mosquitoes that had acquired blood meals from passeriformes each month ranged from 30% to 45% (mean: 38.0%). August is the beginning of the long-distance migration season for many species of passerine birds that migrate from the United States to the Yucatan Peninsula of Mexico. Thus, the increase in the number of mosquitoes feeding on passerines in the latter half of the year could have been due to an increase in the relative abundance of passerines as a consequence of long-distance migration.

The most frequent mammalian hosts for *Cx. quinquefasciatus* were dogs (8.8%) and humans (6.7%) (Table 2). Cats, horses, and pigs were not a common source of blood. The proportion of mosquitoes that had acquired blood meals from dogs was highest in September (25%) and November (20%) (Table 3). No blood meals were obtained from mammals in December.

**Table 1.**
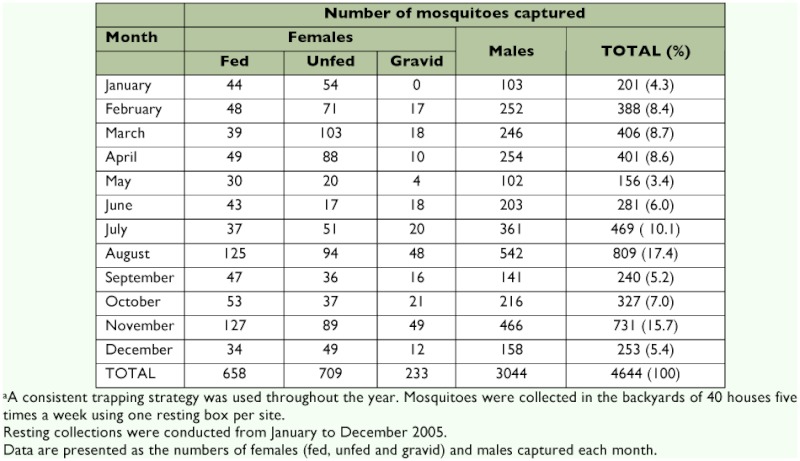
Numbers of *Culex quinquefasciatus* captured outdoors (in the backyard of houses) each month in Merida, Yucatan State.

**Table 2.**
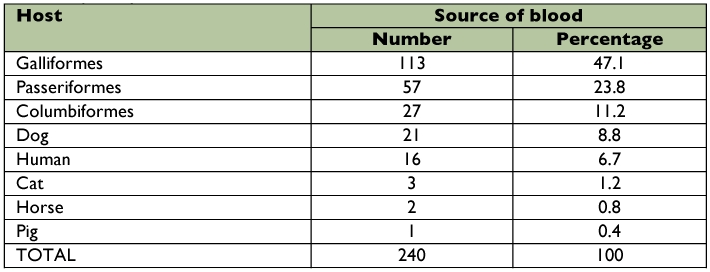
The proportion of blood meals taken from different avian orders and mammalian species by *Culex quinquefasciatus* in Merida, Yucatan State from January to December, 2005.

**Table 3.**
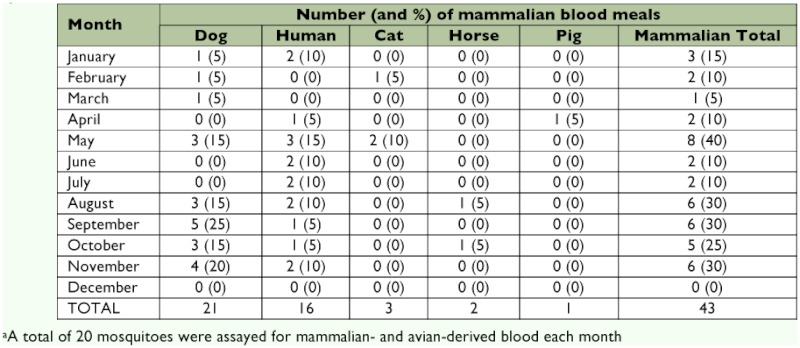
The source of mammalian-derived blood meals from engorged *Culex quinquefasciatus* captured in Merida. DNA extracted from mammalian-derived blood meals was examined by restriction enzyme digestion to identify the host to the species level^a^.

One limitation of this study is that a subset of mosquitoes could have acquired blood meals from vertebrate hosts that were located in nearby houses and therefore were not included in the census. Mark-release-recapture studies have shown that *Cx. quinquefasciatus* can travel up to 1.0 km/day to acquire blood meals (Reisen et al. 1991). However, the census was restricted to the 40 houses used for the mosquito collections because mosquitoes are more likely to feed on vertebrate hosts in their immediate vicinity and because of the logistical restraints associated with censusing all vertebrate hosts within the *Cx. quinquefasciatus* flight path.

### Human blood index and forage ratio values

The overall HBI was 6.7% (Table 2). On five occasions, the monthly HBI values were 10%. The highest monthly HBI values occurred in May (15%), and three months were 0% (February, March, and December) (Table 3). The numbers of humans and domestic animals (mammals and birds), residing at each house sampled in this study were counted. Overall, 88 (41%)) of the vertebrates were human, 45 (21%) were chickens and turkeys (galliformes), 32 (15%) were dogs, 14 (7%) were cats, 5 (3%) were passeriformes and 4 (2%) were horses. There were no pigs, cows or columbiformes Humans were the most common vertebrate species in the study area. However, the FR for humans was < 1.0, indicating that *Cx. quinquefasciatus* had a preference for other vertebrate hosts (Table 4). Forage ratios were calculated for other vertebrate hosts; and those with FRs > 1.0 were Passeriformes (9.1) and Galliformes (2.0) birds.

### Host feeding indices

Calculation of the HFIs for each pair of vertebrate hosts revealed that humans were the least preferred source of blood relative to species of Passeriformes, and Galliformes birds, and dogs, horses and cats (Table 5). HFI values could not be calculated for Columbiformes or pigs because none were present at any study sites. In congruence with the FR values, the HFI data suggested that Passeriformes were the preferred source of blood for *Cx. quinquefasciatus,* followed by Galliformes.

**Table 4.**
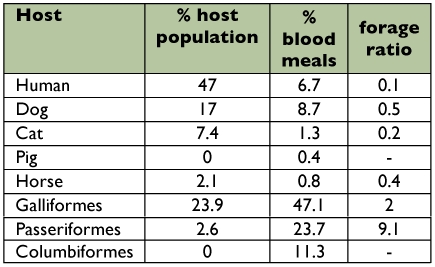
Forage ratios for *Culex quinquefasciatus* mosquitoes in Merida, Yucatan State.

**Table 5.**
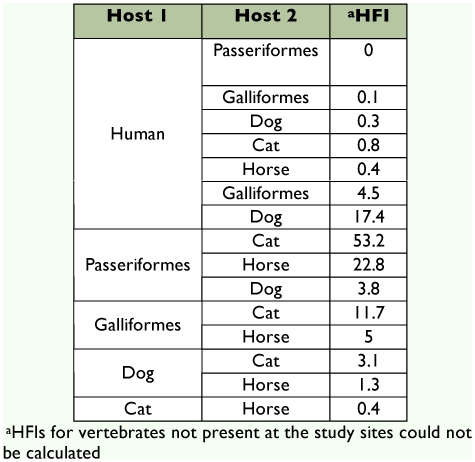
Host feeding indices for each pair of vertebrate hosts fed upon by *Culex quinquefasciatus* in Merida, Yucatan State.

### Summary

This study suggests that *Cx. quinquefasciatus* populations in Merida, Yucatan State feed most frequently on birds year-round. These findings suggest that *Cx. quinquefasciatus* could be an important amplification vector of WNV in this region. In addition, the data refute the hypothesis that the low incidence of WNV illness in Mexico is due to the lack of interaction between *Culex* spp. mosquitoes and avian reservoir hosts in this region. Ornithophilic feeding behavior also was reported recently for *Cx. quinquefasciatus* populations in the city of Monterrey, northern Mexico; 44% to 73% of engorged mosquitoes collected outdoors had acquired their blood meals from chickens (Elizondo-Quiroga et al. 2006). The overall HBI in the present study was low (0.1) indicating that humans were not a preferred host for *Cx. quinquefasciatus* in Merida. Nevertheless, a small proportion (6.7%)) of engorged *Cx. quinquefasciatus* contained human blood, indicating that this mosquito species could also transmit WNV to humans in this region. Vector competence studies are needed to determine the role that *Cx. quinquefasciatus* plays in the transmission of WNV in Mexico.

## Abbreviations

WNV: West Nile virus,
HBI: human blood index,
FR: forage ratio,
HFI: host feeding index
